# Optimization of Dental Devices and Tools Used on Teeth

**DOI:** 10.1155/2021/9913788

**Published:** 2021-08-06

**Authors:** Berna Uzun, Dilber Uzun Ozsahin, Hani Hamdan, Jinan Charafeddine, Gürkan Ünsal, Deniz Özyer

**Affiliations:** ^1^Near East University, DESAM Research Institute, Nicosia, TRNC, Mersin 10, Turkey; ^2^Near East University, Department of Mathematics, Nicosia, TRNC, Mersin 10, Turkey; ^3^Near East University, Department of Biomedical Engineering, Nicosia, TRNC, Mersin 10, Turkey; ^4^University of Sharjah, College of Health Science, Medical Diagnostic Imaging Department, Sharjah, UAE; ^5^Université Paris-Saclay, CentraleSupélec, CNRS, Laboratoire des Signaux et Systèmes (L2S UMR CNRS 8506), Gif-sur-Yvette, France; ^6^Université Paris-Saclay, Laboratoire d'Ingénierie des Systèmes de Versailles (LISV EA4048), Vélizy, France; ^7^Near East University, Faculty of Dentistry, Department of Dentomaxillofacial Radiology, Nicosia, TRNC, Mersin 10, Turkey

## Abstract

Taking decisions is important in every aspect of life. Decision-making has become a difficult problem in any situation where there are multiple criteria. The application of multicriteria decision-making methods that can bring mathematical and logical solutions to the problem from an analytical perspective has experienced considerable growth recently. It provides great benefits in solution and subsequent stages. Medical equipment selection is also a challenging, complex, and difficult problem for the decision-maker, due to the requirements of conflicting criteria, which must be taken into account simultaneously. In this context, the aim of this study implicates the principle of multicriteria decision-making theories on various types of instruments used in dentistry. Since the data used in this study are not numeric but linguistic, the Fuzzy PROMETHEE decision-making method is used. In this research, six dental tools most commonly used by professionals to perform operations on patients are compared and evaluated. Fuzzy PROMETHEE decision-making method investigations show that the dental mirror is the most effective tool among all compared tools, followed by dental suction, dental air abrasion, dental handpiece, dental laser, and dental X-ray, consequently, basing on the selected criteria and the importance weight given to each criterion. Using this technique, one can obtain more specific ranking results based on a specific preference level.

## 1. Introduction

Dental health is a critical factor in determining one's quality of life. The teeth are the only bone tissue in the human body that is not covered by skin. Subsequently, they are the most sensitive bone tissue in the body. Dentists often face difficulties intervening in the disease of the oral cavity. The tools used are critical and important in diagnosing dental caries, periodontal diseases, oral cancers, oral manifestations of HIV, oro-dental trauma, cleft lip and palate, and noma (severe gangrenous disease starting in the mouth, mostly affecting children).

These devices may or may not be electronic. They enable a doctor or a dentist to detect health problems and determine what kind of treatment is to be indicated. However, there are a big variety of medical devices and tools, as well as many types of illnesses. Therefore, this study focuses on a few dental problems and then discusses six types of mostly used and easily accessible dental tools and devices,with their advantages and disadvantages. In this research, the focus is on the use of medical devices for dental pathologies. Also, this research covers those devices that can be used in hospitals and dental clinics.

The focus of this paper is to evaluate the benefits of dental tools/devices and how they make dental treatments easier and more efficient. This paper will explain certain treatments, followed by a discussion on some tools and devices used in dentistry. Finally, this study will provide insight into the future of dentistry equipment and how it can be used to provide services that will make patients happy when they leave the dentist's office. This equipment is composed of several components, some of which have removable heads; it is critical to understand how to replace those components. During the majority of procedures (i.e., operation), the majority of these devices, such as suction, must be used continuously.

The decision-making process is the ability of the decision-makers to choose the most suitable alternative for their purposes and according to determined criteria. Multicriteria decision-making (MCDM) is a subbranch of the decision-making process. There are different types of MCDM techniques available for different types of decision matrix, which contains the parameters of the alternatives, and it becomes more complicated when conflicting criteria occur. These techniques are not applicable where there is uncertain information arising in decision problems. In such cases, fuzzy modeling enables the decision-maker to define the problem mathematically and obtain a solution. However, the comparison of fuzzy numbers is a complex part of the fuzzy MCDM problems. Additionally, it contains the most important part of these problems. The selection problems mostly contain uncertain parameters; therefore, defining the problem by considering the uncertainties requires fuzzy modeling and comparison between the fuzzy sets. There are numerous types of fuzzy MCDM modeling methods available for the comparison of alternatives in a fuzzy environment [1–3]. Yager first presented the centroid method for comparing and ranking fuzzy numbers, even though this technique was known for a hundred years [4]. After Yager's study, different types of ranking indices of the fuzzy sets have been proposed by numerous studies [5–7]. Dong and Wan proposed a new method of PROMETHEE-fuzzy linear programming to give a rational solution to multiattribute group decision-making problems where the decision matrix contains heterogeneous information such as real numbers, intuitionistic fuzzy sets, intervals, triangular intuitionistic fuzzy numbers, and trapezoidal fuzzy numbers, and where the importance weight is not precise [8]. Wan et al. defined the left-right geometric consistency of the triangular multiplicative preference relation by including the experts' trust level in the model and provided a new decision-making method for individuals and for the group in order to extract the experts' weights [9]. Dong et al. proposed the best-worst method for the optimistic and pessimistic decision-maker to define the fuzzy weight vector for the multicriteria decision problems with the fuzzy consistency index [10]. Wan and Dong gave detailed information on interval-valued intuitionistic fuzzy sets and their application in decision-making theories [11]. Wan et al. provided the left-right geometric consistency definition of the triangular multiplicative preference relations (TRMF) by considering the trust levels of the experts in order to create a model with an acceptable consistent TMPR, and that study has proposed two algorithms for the individual and group decision-making problems [12]. Those studies are also beneficial for the comparison of fuzzy numbers. However, the strength of the Yager index for the optimization of fuzzy sets is also shown [13].

In this paper, the Fuzzy PROMETHEE technique, which uses multicriteria decision-making for choosing correct dental device characteristics, is implemented.

### 1.1. Dental Mirror

A dental mirror is a probe with a thin circular mirrored surface on the working end that allows the dentist to see the inside of the teeth and the rest of the oral cavity. This instrument is used to search for bacteria, cavities, and calculus in hard-to-see areas of the mouth. A dental mirror costs about $10. Dentists use three different types of mirrors: front surface, concave, and plane surface. Since it offers a clear image, the front surface mirror is the most frequently used dental mirror [14].

### 1.2. Dental Probe (Dental Handpiece)

A dental probe is a pointed instrument used by dentists to diagnose dental problems rather than treat them. A dental probe is a hand-held instrument with a curved end and a blunt, pointed tip. It is used to diagnose and assess dental diseases and conditions. There are many styles of explorers, including straight, interproximal, and curved. A dental probe is around $200 [15]. Dental probes are used to determine the depth of periodontal pockets formed by teeth. Periodontal depth is critical since abscessed teeth are often associated with advanced periodontal disease, necessitating gum surgery. Therefore, it is important to check the margin regularly to identify any issues before they become significant and the tooth cannot be saved [16].

### 1.3. Dental Laser

Although they are relatively new, dental lasers are already being used to treat a range of conditions, including soft and hard tissue, gums and teeth, caries detection, biostimulation, low-level laser therapy, and photostimulation. Dentists use this device to remove diseased dental tissue and prepare the tooth for dental reconstruction. Dental lasers are used by some dentists to treat conditions such as tooth decay, gum disease, biopsy, and teeth whitening. As is the case for many devices, this one has several advantages and disadvantages. However, since the dental laser is a relatively new method, it has created controversy in the dental community. Several benefits include decreased physical discomfort and anxiety for the patient. When the laser was used instead of more conventional methods, less bleeding was detected during operations. Some drawbacks include the fact that it is more expensive than other tools. It is over $1000 in price [17].

### 1.4. Dental Suction

Dental suctions, also known as saliva ejectors, are used by dentists to remove excess saliva from patients' mouths during care. Additionally, dentists use the system to remove any unnecessary dental materials. Teeth must remain clean, dry, and free of blood, saliva, and water. If the patient has saliva or dental materials in his or her mouth, the operation may be delayed. Because it is a dental appliance, it is difficult to clean after each patient. Rather than that, it is simpler to replace it with a sterile tip for the next patient. These tips are frequently made of plastic or metal. The tips can be disposable or autoclavable, which requires 15 to 20 minutes to complete the sterilization cycle. Since certain procedures can cause the patient to gag, which may result in errors, dentists may use this device for the patient's comfort. Dental suction costs about $600 [18].

### 1.5. Dental Air Abrasion

Dental air abrasion is one of the most costly pieces of equipment reviewed in this study. It costs approximately $700, while the dental mirror costs about $10. While they are similar in scale, their functions and costs are very different. The dental mirror is used to locate the problem, and dental air abrasion is used to remove decay from the tooth by blowing a stream of aluminum oxide-containing air onto the tooth [19].

### 1.6. Dental X-Ray

X-ray is often used to provide a better view of the teeth and their roots. Dentists may use dental X-rays to detect cavities, their depth, and the presence of any other conditions before initiating treatment. Additionally, it shows the emerging teeth that are still under the gums. Dental X-rays can diagnose oral health issues in patients early on, including oral infections, some types of tumors, and gum disease. The dental X-ray is the most expensive instrument discussed in this article. It is both because it is a large electronic system and because it is essential in dentistry. Dental X-rays have evolved significantly since the early years of the twenty-first century. As a result, it is unsurprising that dental X-rays cost about $800 [20].

## 2. Material and Methodology

### 2.1. PROMETHEE (Preference Ranking Organization Method for Enrichment Evaluation)

The PROMETHEE (Preference Ranking Organization Method for Enrichment Evaluation) method technique developed by Professor Jean-Pierre Brans in 1982 [21, 22] is one of the easiest and most efficient models compared to other multicriteria decision-making methods. The best solution to the problem is obtained by comparing each criterion selected for each device. This method is a fair comparison of variants from the point of view of all criteria step by step. Only two types of parameters are required in the PROMETHEE model: each separate criterion and weights of the criteria.

In this context, using this MCDA (multicriteria decision analysis) technique gained so many advantages. A user-friendly outranking method is easy and has been successfully applied to real-life planning problems. PROMETHEE I and PROMETHEE II give a partial and net ranking of the alternatives, respectively, while still satisfying simplicity [23].

The main process of the PROMETHEE technique is as follows.

After the collection of the decision matrix, each selected criterion *j*, the preference function *p*_*j*_(*d*), and the important degrees of each criterion  (*w*_*k*_) will be determined by the decision-makers. Then, the outranking relation (*π*(*a*_*t*_, *a*_*t*′_)) should be calculated for each pair of alternatives (*a*_*t*_ and *a*_*t*′_ ∈ *A*) using

(1) $$\begin{array}{c} \pi \left(a_{t},a_{t^{\prime }}\right)=\overset{K}{\underset{k=1}{\sum }}w_{k}.\left[p_{k}\left(f_{k}\left(a_{t}\right)-f_{k}\left(a_{t^{\prime }}\right)\right)\right],AXA⟶\left[0,1\right]. \end{array}$$

*π*(*a*, *b*) denotes the preference index where *k* denotes the *k*-th criteria. This index shows the preference intensity of *a*_*t*_ compared to *a*_*t*′_ by considering each criterion simultaneously.

Then, the positive outranking flow (*Φ*^+^(*a*_*t*_)) and negative outranking flow (*Φ*^−^(*a*_*t*_)) should be counted by using Equations (2) and (3) sequentially.

- The positive outranking flow of alternative *a*_*t*_:

(2) $$\begin{array}{c} \Phi^{+}\left(a_{t}\right)=\frac{1}{n-1}\overset{n}{\underset{\begin{array}{c} t^{\prime }=1\\ t^{\prime }\neq t \end{array}}{\sum }}\pi \left(a_{t},a_{t^{\prime }}\right). \end{array}$$

(ii) The negative outranking flow of alternative *a*_*t*_:

(3) $$\begin{array}{c} \Phi^{-}\left(a_{t}\right)=\frac{1}{n-1}\overset{n}{\underset{\begin{array}{c} t^{\prime }=1\\ t^{\prime }\neq t \end{array}}{\sum }}\pi \left(a_{t^{\prime }},a_{t}\right), \end{array}$$

where *n* denotes the number of alternatives.

The positive outranking flow is the degree of dominating other alternatives while the negative outranking flow is the degree of being dominated by the other alternatives. Therefore, based on the positive and negative outranking flows, the partial preorder of the alternatives can be obtained based on the following cases:

**Case 1 .**

Alternative *a*_*t*_ should be preferred to the alternative *a*_*t*′_(*a*_*t*_*Pa*_*t*′_) if

(4) $$\begin{array}{c} \left\{\begin{array}{c} \Phi^{+}\left(a_{t}\right)>\Phi^{+}\left(a_{t^{\prime }}\right),\Phi^{-}\left(a_{t}\right)\leq \Phi^{-}\left(a_{t^{\prime }}\right),\\ \Phi^{+}\left(a_{t}\right)=\Phi^{+}\left(a_{t^{\prime }}\right),\Phi^{-}\left(a_{t}\right)<\Phi^{-}\left(a_{t^{\prime }}\right). \end{array}\;\right. \end{array}$$

**Case 2 .**

*a*_*t*_ is not different from *a*_*t*′_(*a*_*t*_*Ia*_*t*′_) if

(5) $$\begin{array}{c} \left(a_{t}Ia_{t^{\prime }}\right)\text{ if}:\Phi^{+}\left(a_{t}\right)=\Phi^{+}\left(a_{t^{\prime }}\right),\Phi^{-}\left(a_{t}\right)=\Phi^{-}\left(a_{t^{\prime }}\right). \end{array}$$

**Case 3 .**

*a*_*t*_ is incomparable to *a*_*t*′_(*a*_*t*_*Ra*_*t*′_) if

(6) $$\begin{array}{c} \left\{\begin{array}{c} \Phi^{+}\left(a_{t}\right)>\Phi^{+}\left(a_{t^{\prime }}\right),\Phi^{-}\left(a_{t}\right)>\Phi^{-}\left(a_{t^{\prime }}\right),\\ \Phi^{+}\left(a_{t}\right)<\Phi^{+}\left(a_{t^{\prime }}\right),\Phi^{-}\left(a_{t}\right)<\Phi^{-}\left(a_{t^{\prime }}\right). \end{array}\right. \end{array}$$

If the 3^rd^ case occurs during partial preorder calculation, applyed to the PROMETHEE II, the net outranking values (*Φ*^net^(*a*_*t*_)) should be calculated using

(7) $$\begin{array}{c} \Phi^{\text{net}}\left(a_{t}\right)=\Phi^{+}\left(a_{t}\right)-\Phi^{-}\left(a_{t}\right). \end{array}$$

And the net ranking results of the alternatives should be determined based on the following cases:

**Case 4 .**

*a*_*t*_ is preferred to

(8) $$\begin{array}{c} a_{t^{\prime }}\left(a_{t}Pa_{t^{\prime }}\right)\text{ if }\Phi^{\text{net}}\left(a_{t}\right)>\Phi^{\text{net}}\left(a_{t^{\prime }}\right). \end{array}$$

**Case 5 .**

*a*_*t*_ is not different than

(9) $$\begin{array}{c} a_{t^{\prime }}\left(a_{t}Ia_{t^{\prime }}\right)\text{if }\Phi^{\text{net}}\left(a_{t}\right)=\Phi^{\text{net}}\left(a_{t^{\prime }}\right). \end{array}$$

The most preferable alternative should have a higher net outranking flow.

### 2.2. Fuzzy PROMETHEE (F-PROMETHEE)

Fuzzy logic is a multivalued logic that describes vague conditions more rationally [24, 25]. Real-life problems often involve vague situations that are often difficult to quantify numerically and can only be expressed linguistically. The Fuzzy PROMETHEE (F-PROMETHEE) method has been developed as a hybrid model because of the insufficient PROMETHEE method in such problems. The main aim of the Fuzzy PROMETHEE model is to propose a comparison between two fuzzy sets. There have been few types of research that applied Fuzzy PROMETHEE to make an optimal decision on different realistic problems such as cancer treatment techniques, nuclear medicine and oncology, analysis of image reconstruction, and X-ray-based medical imaging devices [23, 26–31]. Therefore, in this study, the F-PROMETHEE method is used to ensure that the input data are interpreted correctly.

## 3. Results

During the first evaluation, the six most frequently used dental devices (such as dental mirror, dental laser, dental suction, dental air abrasion, dental X-ray, and dental probe) were evaluated and compared using the Fuzzy PROMETHEE decision-making method. These criteria include cost, calibration, period, practicality, advantages, disadvantages, comfortability, dose, and size, as shown in Table 1.

The simulation of devices carried out shows the advantages and disadvantages of these devices according to different criteria. The result shows that the devices have different points on the scale. These devices have different positive and negative values. For example, the cost of a dental mirror is approximately $10, while a dental probe is around $200. While they have different purposes, a dental mirror is cheaper. The dental laser is used for treatments, while the dental mirror is used for diagnosis. Also, the dental mirror does not have anything mechanical or electrical while the dental laser does. Thus, it makes sense for the dental laser to cost so much compared to the dental mirror. The dental mirror is used to identify medical problems, while a dental air abrasion is used to remove decay from the tooth by blowing a stream of aluminum oxide as air into the tooth. While the dental mirror gives immediate results and is temporary, the dental X-ray gives a more permanent image that can be observed for a longer period without making the patient tired. While the dental probe costs approximately $200, dental air abrasion costs about $700. Both of these have a curvy end; However, the dental air abrasion has an end that has a tiny hole, enabling it to spray particles onto the tooth. Instead of that, the dental probe has a sharp end.

Since the data collected for the analysis of dental devices are not numerical, the linguistic fuzzy scale has been used to convert the obtained dataset to numerical values. As a result, the linguistic fuzzy scale is used as shown in Table 2.

The Yager Index (YI = 3*n* − *a* + (*b*/3)) of the fuzzy number $\tilde{F}=\left(n-a,n,n+b\right)$ is applied to defuzzify the linguistic data.

In this study, the input data are also treated as fuzzy numbers. This took into account the uncertainty contained in the data, which could give more valuable ranking results by considering the fuzziness. After the defuzzification, the PROMETHEE process is then employed for the ranking of dental devices with minimizing cost, disadvantages, dose, and size of the alternatives and maximizing the advantages and comfortability. In this analysis, the calibration period and practicability are not used as criteria since there is no difference between the alternatives corresponding to these criteria. Also, the importance weight of the criteria has been chosen equally.

According to the ranking result, the dental mirror is the most effective tool among others as shown in Table 3. These results were obtained on the basis of the criteria. The complete ranking is obtained according to the selected weights. According to Table 3, the best solutions are those with the highest net flow. Table 3 shows the complete ranking of dental devices according to the selected criterion, which is necessary for the performance of the devices. At least, these simulations of filling devices are important and effective for the dental industry.

To test the results obtained with Fuzzy PROMETHEE for the evaluation of the dental devices, the fuzzy TOPSIS (Technique for Order of Preference by Similarity to Ideal Solution) technique is used, which is also an another successfully used MCDA technique. The TOPSIS technique has been defined by Hwang and Yoon in 1981 [30]. It evaluates the alternatives based on their distance to the positive ideal solution (*A*^+^) and the negative ideal solution (*A*^−^) under conflicting criteria. The positive ideal solution is the combination of the best values of each criterion, while the negative ideal solution is the combination of the worst values of each criterion. It applies to the selection problems with the numerical dataset. Furthermore, relative closeness to the positive ideal solution (*R*_*i*_) should be considered as the most preferred alternative with this technique. This is a ratio that can be counted based on the distance of alternatives to the positive ideal solution (*d*^+^) and distance of the alternatives to negative ideal solution (*d*^−^) based on

(10) $$\begin{array}{c} R_{i}=\frac{d_{i}^{-}}{d_{i}^{-}+d_{i}^{+}} \end{array}$$

After demulsifying the data of the dental devices and normalizing the decision matrix, the weighted normalized matrix is computed, and then the positive and the negative ideal solutions are obtained as shown in Table 4.

And the ranking results of the dental devices using the fuzzy TOPSIS method are obtained as shown in Table 5.

The results show that the dental mirror should be the most preferred dental device, followed by dental suction. Additionally, there is a slight change between the rankings of the last two alternatives. Apart from this, the same ranking results have been obtained using the fuzzy TOPSIS technique, which shows the consistency between the ranking results.

This study analyzes and compares the most commonly used fundamental dental tools which are necessary to perform operations on patients, and it shows the strengths and the weaknesses of each alternative device, which will be beneficial for nonexperts or freshly graduated dentists. With different importance levels of the parameters, the individual ranking results could be obtained simply for the specific aim.

## 4. Conclusion

With Fuzzy PROMETHEE, distorted and imprecise inputs, such as the linguistic data used in this analysis, can be compared and evaluated. Due to its resemblance to human reasoning, Fuzzy PROMETHEE can also overcome more complex decision-making problems, which is advantageous for clinical problems such as dental tools collection.

Parameters such as price, calibration, length of use, practicability, advantages, disadvantages, comfortability, dosage, and instrument size, all play a role in the selection process. The study's findings indicate that the dental mirror should be the most preferred dental device, followed by dental suction. Furthermore, there is a small change in the rankings of the two final alternatives. Given that the same rankings were discovered using the fuzzy TOPSIS technique, there was some degree of consistency in the ranking results.

This study sheds light on dental device selection problem, by offering simple and alternative solutions, providing an effective, fast, and practical way to make decisions on this problem by applying a decision-making process. The implementation of the multicriteria decision-making process, which can generate mathematical and logical solutions to problems from an empirical standpoint, offers significant benefits.

## Figures and Tables

**Table 1:** Simulation of dental devices.

	Dental device	Cost ($)	Calibration period	Practicality	Advantages	Disadvantages	Comfortability	Dose	Size
1	Dental mirror	10	Daily	Yes	Medium	Low	Comfortable	None	Low
2	Dental handpiece	200	Daily	Yes	Medium	Medium	Comfortable	Low	Low
3	Dental laser	500	Daily	Yes	High	Medium	Comfortable	Medium	Medium
4	Dental suction	600	Daily	Yes	High	Very low	Comfortable	None	Low
5	Dental air abrasion	700	Daily	Yes	Medium	Low	Comfortable	Very low	Low
6	Dental X-ray	800	Daily	Yes	Very high	High	Comfortable	Low	High

**Table 2:** Selected fuzzy scale for the linguistic data.

Linguistic scale for evaluation	Triangular fuzzy scale
Very high (VH)	(0.75,1, 1)
Important (H)	(0.50,0.75,1)
Medium (M)	(0.25,0.50,0.75)
Low (L)	(0,0.25,0.50)
Very low (VL)	(0, 0, 0.25)

**Table 3:** Fuzzy PROMETHEE ranking results.

Complete ranking	Dental device	Rank		
		*Φ*	*Φ* ^+^	*Φ* ^−^
1	Dental mirror	0.0076	0.0079	0.0003
2	Dental suction	0.0056	0.0063	0.0007
3	Dental air abrasion	0.0039	0.0054	0.0016
4	Dental handpiece	-0.0003	0.0034	0.0037
5	Dental laser	-0.0056	0.0007	0.0063
6	Dental X-ray	-0.0111	0.0007	0.0118

**Table 4:** Positive and negative ideal solutions of dental devices.

Criteria	Aim min/max	*A*^+^/*A*^−^
Cost ($)	Min	0.0078/0.0902
Advantages	Max	0.0929/0.0505
Disadvantages	Min	0.0122/0.1144
Comfortability	Max	0.0962/0.0000
Dose	Min	0.0000/0.1349
Size	Min	0.0404/0.1213

**Table 5:** Fuzzy TOPSIS ranking results.

Ranking	Alternatives	*d*^+^/*d*^−^	*R*_*i*_
1	Dental mirror	0.0497/0.2159	0.8128
2	Dental suction	0.0679/0.2130	0.7582
3	Dental air abrasion	0.0986/0.1856	0.6530
4	Dental handpiece	0.1414/0.1299	0.4787
5	Dental X-ray	0.1939/0.0797	0.2914
6	Dental laser	0.1945/0.0633	0.2455

## Data Availability

The fuzzy data used to support the findings of this study are available from the corresponding author upon request.
